# Support vector machine prediction of enzyme function with conjoint triad feature and hierarchical context

**DOI:** 10.1186/1752-0509-5-S1-S6

**Published:** 2011-06-20

**Authors:** Yong-Cui Wang, Yong Wang, Zhi-Xia Yang, Nai-Yang Deng

**Affiliations:** 1 College of Science, China Agricultural University, Beijing, China, 100083; 2 Key Laboratory of Adaptation and Evolution of Plateau Biota, Northwest Institute of Plateau Biology, Chinese Academy of Sciences, Xining, China, 810001; 3 Academy of Mathematics and Systems Science, Chinese Academy of Sciences, Beijing, China, 100190; 4 College of Mathematics and System Science, Xinjiang University, Urumuchi, China, 830046

## Abstract

**Background:**

Enzymes are known as the largest class of proteins and their functions are usually annotated by the Enzyme Commission (EC), which uses a hierarchy structure, i.e., four numbers separated by periods, to classify the function of enzymes. Automatically categorizing enzyme into the EC hierarchy is crucial to understand its specific molecular mechanism.

**Results:**

In this paper, we introduce two key improvements in predicting enzyme function within the machine learning framework. One is to introduce the efficient sequence encoding methods for representing given proteins. The second one is to develop a structure-based prediction method with low computational complexity. In particular, we propose to use the conjoint triad feature (CTF) to represent the given protein sequences by considering not only the composition of amino acids but also the neighbor relationships in the sequence. Then we develop a support vector machine (SVM)-based method, named as SVMHL (SVM for hierarchy labels), to output enzyme function by fully considering the hierarchical structure of EC. The experimental results show that our SVMHL with the CTF outperforms SVMHL with the amino acid composition (AAC) feature both in predictive accuracy and Matthew’s correlation coefficient (MCC). In addition, SVMHL with the CTF obtains the accuracy and MCC ranging from 81% to 98% and 0*.*82 to 0*.*98 when predicting the first three EC digits on a low-homologous enzyme dataset. We further demonstrate that our method outperforms the methods which do not take account of hierarchical relationship among enzyme categories and alternative methods which incorporate prior knowledge about inter-class relationships.

**Conclusions:**

Our structure-based prediction model, SVMHL with the CTF, reduces the computational complexity and outperforms the alternative approaches in enzyme function prediction. Therefore our new method will be a useful tool for enzyme function prediction community.

## Background

Enzymes are known as the cellular machines that can catalyze chemical reactions and convert the molecules called substrates into different molecules called the products. Almost all processes in a biological cell need enzymes. So it is known that enzymes are the largest and one of the most important families in the proteins. It was estimated that about half of all the proteins have been characterized as function of enzymatic activity by various biochemical experiments. Therefore, accurate assignment of enzyme function is crucially important and is a prerequisite of high-quality metabolic reconstruction and the analysis of metabolic fluxes [1].

One great effort for enzyme study is from the International Commission on Enzymes to annotate the function of enzymes by the Enzyme Commission (EC) number, which is a numerical classification scheme to distinguish enzymes by the enzyme-catalyzed reactions. The EC number has a hierarchy structure and is comprised of four integers separated by periods to classify the functions of enzymes [2]. The first three digits describe the overall type of an enzymatic reaction, while the last digit represents the substrate specificity of the catalyzed reaction. Since the first three digits have the direct relationship with enzyme function, it is in pressing need to develop computational methods to accurately predict the first three EC digits for a given protein.

The straightforward idea is to transfer an EC number between two globally similar protein sequences. However, this method only works well when two sequences are very similar. The accuracy of the direct inference has been reported to significantly drop under 60% sequence identity [3]. Other efforts are trying to develop computational methods to automatically categorize enzyme into EC hierarchy in a machine leaning framework. For example, some works predict the top layer of the EC taxonomy, and then output the second and third digits of EC number, respectively. For example, Chou et.al [4] developed a top-down approach for predicting enzyme functional classes and sub-classes, and the overall accuracy for the first two layers is higher than 90%. However, they treated functional class independently, and ignored the inter-class relationships. In this paper, we develop a support vector machine (SVM)-based methods which takes hierarchical structure of enzyme functional labels into account. With this additional information, the improvement of accuracy can be expected. In particular, our method is accomplished by using a generation of the SVM-based discriminant functions that decompose into contributions from different levels of the hierarchy [5]. Importantly, we reformulate the previously proposed structure-based model to make it computational feasible on a large scale dataset by replacing large number of complicated inequalities with one simple inequality. We named our simplified model as the SVMHL (SVM for hierarchy labels). Another key problem in predicting enzyme function is how to encode a protein as a real-value vector. In previous studies, the amino acid composition (AAC) representation has been widely utilized in predicting enzyme family and subfamily class [6,7]. The AAC feature representation is denoted by a 20-dimensional vector which consists of occurrence frequencies of single amino acid. However, the catalytic residues always reside in the protein sequence with some particular neighbor amino acids. For example, catalytic residue database [8] contains 178 enzymes and 615 catalytic residues. Among all 178 enzymes, over 80 enzymes have the adjacent catalytic residues. Thus, the sequence encoding features should contain not only the amino acid composition but also the sequence-order information. If the additional neighbor information in sequence is incorporated into the predictive model and better results can be expected. We noticed that some modified versions of AAC consider the sequence-order information, such as pseudo amino acid composition (Pse-AAC) [9] and amphiphilic pseudo-amino acid composition (Am-Pse-AAC) [10]. However, both Pse-AAC and Am-Pse-AAC have introduced some un-determined parameters to consider the physical chemistry properties of amino acids. Recently, a much simple feature encoding method, called conjoint triad feature (CTF), has been proposed for protein-protein interactions (PPIs) [11]. The authors have shown that SVM with the CTF outperforms other sequence-based PPI prediction methods. The CTF considers not only properties of the target amino acid but also its neighbor amino acids and treats any three continuous amino acids as an unit. That is, it contains not only the composition of amino acids but also sequence-order information. Inspired by these, in this paper, we introduce the CTF into our SVMHL to predict enzyme function.

Collectively, we introduce two key improvements in predicting enzyme function within the machine learning framework in this paper. One is to introduce the efficient sequence encoding methods (CTF) for representing given proteins. The second one is to develop a structure-based prediction method (SVMHL) with low computational complexity. Specifically, we present the benchmark dataset collection, the CTF formulation, and the procedure for formulating the structure-based predictive model in Materials and Methods section. In Results section, we compare our method with the existing methods in different perspectives and demonstrate the performance of our new method. Also, the discussions and conclusions are presented for further study.

## Results and Discussions

### Proof-of-concept example for SVMHL

Our model, the SVMHL, is extended from the previous structural leaning method in (5) ~ (7) denoted as PMSVMHL in this paper (See Materials and Methods). Following the idea of Theorem 2, we decrease the number of variables from *l* × (*q -* 1) to *l* by replacing large numbers of inequalities with one simple inequality. To illustrate the improvement of our simplified model-SVMHL, we carry out a proof-of-concept analysis on a simple dataset. This dataset is UCI glass dataset (available at http://www.ics.uci.edu/~mlearn/MLRepository.html). The glass dataset contains two main classes, and each main class can be further classified into subclasses. For convenience, we illustrate the hierarchical structure of this simple dataset in Figure 3(c) . Concretely, we take out 70 float processed building windows and 76 non-float processed building windows, and both of them belong to the window glass. In addition, 29 headlamps which belong to the non-window glass are also included in the training dataset. In total, the toy example contains 175 data points classified into three class, and two classes of them can be further integrated. We leave 9 headlamps, 10 float processed building windows, and 10 non-float processed building windows for testing, and the rest data are for training of the classification rule (Refer to Table 1 for details).

**Table 1:** The statistics of training and testing dataset for the toy example. Class 1 is non-window glass, Class 2 is float processed building window glass, and Class 3 is non-float processed building window glass.

*Dataset type*	*Class1*	*Class2*	*Class3*
*Training set*	20	60	66
*T esting set*	9	10	10

We train the standard SVM, PMSVMHL, and SVMHL on the training dataset, respectively, and apply the corresponding trained classification rules to the testing examples. The test accuracy is shown in Table 2. SVMHL can get the competitive results of PMSVMHL. And both SVMHL and PMSVMHL perform better than the standard SVM. That’s reasonable because both SVMHL and PMSVMHL take the relationship among classes into account, i.e., Class 2 and Class 3 belong to the same super-family. Besides that, the standard SVM, PMSVMHL, and SVMHL are implemented on a PC machine with Intel Core 2 Due CPU 1.60 GHz. The training time for PMSVMHL and SVMHL are 165 seconds and 10 seconds, respectively. The training time of PVSVMHL is about ten times longer than SVMHL. The increased efficiency of our method improves the feasibility of applying our method for predicting enzyme function in large scale. The proof-of-concept study on this simple toy example suggests that SVMHL is a reasonable simplified version of PMSVMHL.

**Table 2:** The predictive accuracy on glass testing set.

Class type	Standard SVM	PMSVMHL	SVMHL
*class1*	100%(9/9)	100%(9/9)	100%(9/9)
*class2*	50%(5/10)	80%(8/10)	80%(8/10)
*class3*	100%(10/10)	100%(10/10)	100%(10/10)
*overall*	82.8%	93.1%	93.1%

### The results on the benchmark enzyme dataset

Then we apply the newly developed method to predict enzyme function in a benchmark dataset collected from the literature [4] (Refer to Materials and Methods for detail). To illustrate the contribution of CTF encoding method and SVMHL, respectively, we designed two experiments. Firstly, we fix the machine leaning method and compare the feature encoding methods. Secondly we fix the feature encoding method and compare the performance of machine learning methods by considering the hierarchy structure of labels. We note that we only report the predictive results on the second and third level of EC hierarchy in the following experiments since there are no inter-class relationships in the top level of EC hierarchy.

#### Comparison with the alternative encoding methods

Since the catalytic residues tend to reside in the protein sequence with their vicinal amino acids together, the sequence features with sequence-order information may bring the significant improvement in enzyme function prediction. To validate this, we compare the performance of SVMHL with the CTF and SVMHL with the AAC. We simply take the Ec1 subset as an example. In Ec1 subset, there are eight enzyme sub-subfamilies in total: Ec1.1.1, Ec1.2.1,Ec1.3.1, Ec1.5.1, Ec1.6.5, Ec1.6.99, Ec1.9.3, and Ec1.11.1. It is obvious that, sub-subfamily Ec1.6.5 and Ec1.6.99 belong to the same subfamily Ec1.6, so in the second level of the EC hierarchy, there are seven functional classes in total.

**Figure 1:**
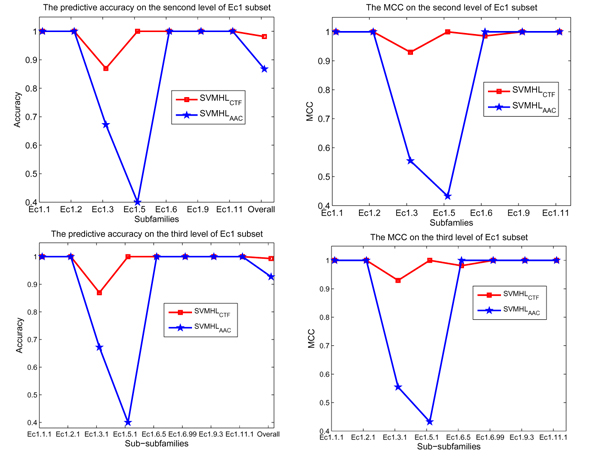
**The comparison of feature encoding methods ACC and CTF**. The predictive accuracy (the left two subfigures) and MCC (the right two subfigures) on the second and third level of Ec1 subset for SVMHL with the CTF (*SVMHL_CTF_*) and SVMHL with the AAC (*SVMHL_AAC_*)*.*

SVMHL with the CTF and SVMHL with the AAC are validated on the Ec1 subset, respectively, the predictive accuracy and Matthew’s correlation coefficient (MCC) on the second and third level of EC hierarchy are plotted in Figure 1. The Figure 1 shows that SVMHL with the CTF outperforms SVMHL with the AAC not only in higher predictive accuracy, but also in higher MCC. The results are robust to the choice of the second and the third levels of EC hierarchy. These results suggest that the CTF sequence feature with sequence-neighbor information brings significant improvement in enzyme function prediction.

#### Comparison with the methods without hierarchy information

Next we would like to show the promising performance of SVMHL with the CTF by training it on a low-homologous enzyme benchmark dataset (Refer to Materials and Methods for detail). It should be noted that predicting enzyme function is an imbalance multi-class classification problem due to the fact that the numbers of proteins in each enzyme family and subfamily are very different. In our previous work [12], we show that the AM-SVM (SVM with arithmetic mean offset) [13], which is specially designed for imbalance classification problem, performs better than the standard SVM with the CTF in predicting enzyme subfamily classes. Therefore, we train the AM-SVM with the CTF on the same benchmark dataset for comparison. The AM-SVM treats each class label independently, and ignores the inter-calss relationships. Since both SVMHL and AM-SVM apply the CTF as the features. We choose to fix the feature encoding methods here for fair comparison.

We compare two methods regarding to the variability of predictive accuracy and MCC for enzyme subfamily classes and sub-subfamily classes. The results are presented in Table 3 and Table 4, respectively. These two tables show that, for the second EC digit (enzyme subfamily class), SVMHL outperforms AM-SVM regarding to the two predictive indexes. Specifically, AM-SVM makes the mean accuracy range from 89% to 95%, while the mean accuracy for SVMHL ranges from 90% and 98%. For the third EC digit (enzyme sub-subfamily class), AM-SVM makes the mean accuracy range from 84% to 96%, while the mean accuracy for SVMHL ranges from 86% and 98%. Therefore, for both the second and third EC digits, SVMHL outperforms AM-SVM not only on the range of accuracy waved but also on the mean of accuracy except for the Ec1 subset.

**Table 3:** The predictive accuracy and MCC on the second level of EC hierarchy for AM-SVM and SVMHL.

Family name	AM-SVM		SVMHL	

	Accuracy(%)	MCC	Accuracy(%)	MCC
*EC*1 : *Oxidoreductases*	95.3 ± 3.8	0.95 ± 2.9%	98.1±4.9	0.98±2.6%
*EC*2 : *Transferases*	94.1 ± 2.9	0.90 ± 8.4%	97.6±2.6	0.93±8.2%
*EC*3 : *Hydrolases*	92.9 ± 3.9	0.91 ± 6.6%	95.4±3.7	0.94±6.3%
*EC*4 : *Lyases*	93.6 ± 9.1	0.93 ± 5.4%	95.8±8.3	0.96±4.7%
*EC*5 : *Isomerase*	94.7 ± 6.4	0.89 ± 4.9%	96.8±6.2	0.92±4.1%
*EC*6 : *Ligases*	89.2 ± 6.9	0.93 ± 5.1%	90.1±6.1	0.96±6.2%

**Table 4:** The predictive accuracy and MCC on the third level of EC hierarchy for AM-SVM and SVMHL.

Family name	AM-SVM		SVMHL	

	Accuracy(%)	MCC	Accuracy(%)	MCC
*EC*1 : *Oxidoreductases*	96.2 ± 4.4	0.96 ± 3.2%	98.3 ± 4.6	0.98±2.4%
*EC*2 : *Transferases*	89.2 ± 9.8	0.91 ± 10.1%	92.1±9.7	0.92±9.5%
*EC*3 : *Hydrolases*	78.9 ± 5.2	0.81 ± 9.7%	81.7±4.9	0.81±8.9%
*EC*4 : *Lyases*	95.6 ± 7.1	0.94 ± 3.4%	96.7±6.7	0.97±2.9%
*EC*5 : *Isomerase*	78.8 ± 4.1	0.89 ± 3.1%	81.3±3.4	0.91±2.7%
*EC*6 : *Ligases*	84.5 ± 7.1	0.87 ± 8.4%	86.4±6.4	0.91±8.7%

These two tables also show that, AM-SVM makes the mean MCC range from 0*.*89 to 0*.*95 for the second EC digit, while the mean MCC for SVMHL ranges from 0*.*92 and 0*.*98. For the third EC digit, AM-SVM makes the mean MCC range from 0*.*81 to 0*.*96, while the mean MCC for SVMHL ranges from 0*.*82 and 0*.*98. Clearly for both the second and third EC digits, SVMHL outperforms AM-SVM not only on the range of MCC but also on the mean of MCC except for the Ec6 subset.

The imbalance property is incorporated into AM-SVM, and the inter-class relationship and imbalance properties are both introduced into SVMHL. Table 3 and Table 4 show that, SVMHL outperforms AM-SVM with not only predictive accuracy but also MCC for both the second and third EC digits. This result suggest that the more information or properties of dataset are incorporated into the predictive model, the better results can be obtained.

### Comparison with other structural learning methods

In this subsection we compare our method with other structure-based learning methods. In theory, the model of PMSVHL is a special version of the Hierarchical Max-Margin Markov (*HM*^3^) [14] by using the zero-one loss. *HM*^3^ has been successfully used in many structured pattern recognition problems, such as document categorization [5], web contend classification [15] and so on. Furthermore, in [16], the authors have introduced *HM*^3^ and Maximum Margin Regression algorithm (MMR) to predict enzyme function. The MMR [17] generated one-class SVM to perform structure-based prediction. The motivation of the MMR is the same as the SVMHL and can be implemented with low cost. On a gold-standard enzyme dataset containing 3090 proteins, MMR achieved the best accuracy for the all individual EC digits. While *HM*^3^ achieved nearly the same results, and most importantly, *HM*^3^ obtained the best F1 scores and the MMR comes close as the second. When predicting sub-subfamilies, *HM*^3^ obtained a 79% F1 score , and 89% when predicting the subfamilies [16]. Like MCC, F1 score can avoid the bias due to the imbalance problem. For comparison, we train our SVMHL on the same gold-standard dataset. As a result, SVMHL with the CTF has obtained over 80% F1 score in predicting enzyme sub-subfamilies, and over 90% F1 score in predicting subfamilies. These results suggest that SVMHL with the CTF has the competitive performance with *HM*^3^, and outperforms the existing low costly structure-based method MMR. Most importantly, the predictive results also imply that the performance of SVMHL comes from the fact that SVMHL takes a better way to take the hierarchical structure of the inter-class relationships into account.

## Discussions

SVMHL is a simplified version of PMSVMHL by replacing the *q* ( *q* is the total number of categories) inequalities with a single inequality. The selected  makes the upper bound of |(w *·* Φ(*x*,*y*)) *-* (w *·* Φ(*x*,*y′*))|,*y′* ≠ *y* be the lowest (see Materials and Methods). However the ideal  should make the loss of |(w *·* Φ(*x*,*y*)) *-* (w *·* Φ(*x*,*y′*))|,*y′* ≠ *y* be the lowest rather than its upper bound. We emphasize that perhaps this is the simplest form to reduce the scale of PMSVMHL model under the requirement of the lowest loss. Surprisingly, SVMHL with the lowest upper bound of loss obtains the competitive performance of PMSVMHL on a toy example. In addition, SVMHL outperforms other alternative approaches, not only the methods without structure properties but also these structure-based models. Comparing other modified versions of PMSVMHL [18], our approach is easy to be understood and has the advantage of simplicity.

One way to further improve the structure-based model is to design efficient and simple feature encoding schemes. Since it is well-known that efficient feature construction is important in determining the performance of a predictive method. Here SVMHL with the CTF obtains the promising predictive results. One important reason is that CTF feature capture the pattern that the catalytic acids tend to reside in the protein sequence with some particular neighbor amino acids. Thus future work can focus on improving feature extracting methods by introducing the sequence features which consider the sequence-order information, such as K-space encoding scheme [19]. Another way to improve the feature construction methods is to integrate more genome and proteome data sources and use efficient kernel methods to fuse heterogeneous information [20]. In addition, we can define a different similarity measure for each data source and thereby incorporate more prior information into the design of the classifier [21].

There is still plenty room for the improvement on the modification of the structure-based predictive model. As mentioned above, the prediction of enzyme family and subfamily class is an imbalance multi-class classification problem. Although we try to consider the imbalance problem into SVMHL by treating  as the class label belonging to the same subfamily of *y_i_*. We are making the corresponding proteins within this class label are the largest (See Materials and Methods). However, the imbalance problem has not been solved in essence. Future work can reformulate the SVM-based model suitable for the imbalance problem, and make the new model contain not only the hierarchical structure of the output labels but also the imbalance property. There are many efficient and simple SVM model specially designed for imbalance classification problem, such as Multisurface Proximal Support Vector Machine Classification [22], and its extended versions: Twin SVM [23] and Nonparallel plane proximal classifier (NPPC) [24].

Since user-friendly and publicly accessible web-servers represent the future direction for developing practically more useful predictors [25], we shall make efforts in our future work to provide a web-server for the method presented in this paper

## Conclusions

In this paper, we propose two machine learning ideas in enzyme function prediction. Firstly, we develop a structure-based method, the SVMHL, to consider the hierarchy structure of the labels to predict enzyme function in EC hierarchy. Secondly, the CTF, which contains not only the composition of amino acids but also the sequence-order information, is introduced to encode given proteins as the real-value vectors. We first show that our simplification to PMSVMHL is efficient by a UCI glass dataset. The experimental results show that SVMHL has the competitive performance with PMSVMHL. In an Ec1 subset, SVMHL with the CTF outperforms SVMHL with the AAC with not only in the high predictive accuracy, but also in high MCC. In addition, when validated on a low-homologous enzyme dataset, SVMHL with the CTF obtains the best MCC and accuracy of 0*.*91 and 0*.*98 in predicting the main families. Furthermore, SVMHL with the CTF obtains over 0*.*92 and 0*.*82 average MCC in predicting subfamilies and sub-subfamilies, respectively. The predictive results suggest that SVMHL with the CTF performs much better than existing methods which do not take account of hierarchical relationship among enzyme categories for predicting the enzyme function in EC hierarchical taxonomy. Also SVMHL displays better predictive performance than the existing low cost structure-based method MMR on a gold-standard enzyme dataset. Therefore we conclude that our new method holds the potential as a useful supplementary tool for the future studies in enzyme function.

## Materials and Methods

In this section, we describe the collection of benchmark datasets and the construction of the predictive model.

### Materials

The benchmark dataset is collected from the literature [4] to validate the performance of our method. The sequences in this dataset have less than 40% sequence identity to any other sequences in the same functional class. The detail information of this dataset can be found in [4]. In addition, those sub-subfamilies which contain less than 50 proteins are excluded in our validation to avoid the extreme sub-subfamily bias. Finally there are six main functional classes and eighty-five sub-subfamily classes (i.e., eight for oxidoreductases, seventeen for transferases, eighteen for hydrolases, eight for lyases, eight for isomerases, and six for ligases) in the benchmark dataset.

### Methods

#### Input feature: CTF

**Figure 2:**
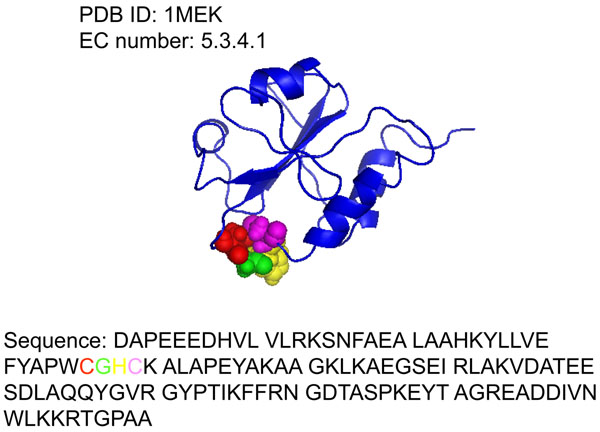
**The protein structure of the enzyme 1MEK and its catalytic residues**. The protein structure of the enzyme 1MEK shows the importance to consider the neighbor amino acids in predicting enzyme function. The colored spheres in the structure and the colored residues in the sequence represent the catalytic residues. 1MEK contains the adjacent catalytic residues in its A chain: Cys (C: 36), Gly (G: 37), His (H: 38), Cys (C: 39). The number in the brackets represents the abbreviation of the residues and its positions in the protein sequence.

Sequence-based prediction is based on the assumption that knowledge of the amino acid sequence alone might be sufficient to estimate the evolutionary history, overall structure and function, and the interacting propensity between two proteins. The reason of enzyme function prediction based only on sequence information is due to the easily available sequence data. To fully take advantage of sequence information, the most important challenge is to find a suitable way to fully describe the important information of protein. To this end, we manually checked the catalytic residue database [8] which contains 178 enzymes and 615 catalytic residues. We found that there are over 80 enzymes have the adjacent catalytic residues among all 178 enzymes. For example, the enzyme 1MEK (EC 5.3.4.1, its structure is shown in Figure 2) has four catalytic residues in its A chain: Cys (C), Gly (G), His (H), Cys (C) locate in 36, 37, 38, 39 position, respectively. These four residues are neighbors in the sequence (highlighted in different colors in Figure 2) and work together for the enzyme function. This indicates that the catalytic residues tend to reside in the protein sequence with some neighbor catalytic residues together.

Based on the above observations, we applied a descriptor named conjoint triad which has been successfully proposed for predicting protein protein interactions. The feature encoding method considers the properties of one amino acid and its vicinal amino acids and regards any three continuous amino acids as a unit. Further, the triads can be categorized according to the classes of amino acids, i.e., triads composed by three amino acids belonging to the same classes could be treated identically, because they may play similar roles in performing enzyme function. Specifically, the 20 amino acids can be classified into seven classes: {*A*, *G*, *V*}, {*I*, *L*, *F*, *P*}, {*Y*, *M*, *T*, *S*}, {*H*, *N*, *Q*, *W*}, {*R*, *K*}, {*D*, *E*}, {*C*} based on the dipoles and volumes of the side chains. Thus, a 343(7 × 7 × 7)-dimension vector is used to represent a given protein, where each element of this vector is the frequency of the corresponding conjoint triad appearing in the protein sequence. More detailed description for the CTF can be found in [11]. In this way, the sequence features which incorporate not only the amino acid composition but also the sequence-order information. In addition, the CTF can be implemented in a economic way and contains no pre-defined parameters.

#### Structure-based prediction model: the SVMHL

**Figure 3:**
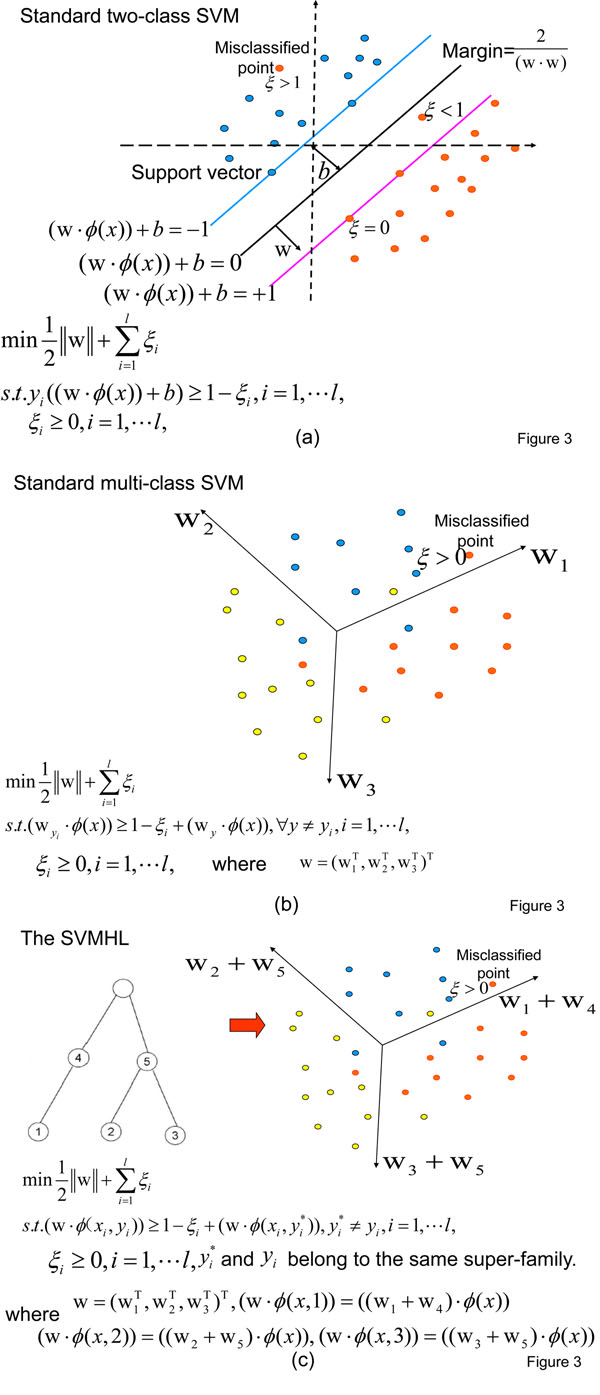
**The scheme of SVMHL and comparison with standard two-class and multi-class SVMs**. The illustration of modeling procedure of the standard two-class SVM (a), the standard multi-class SVM (b), and the SVMHL (c), respectively. *ϕ*:*R^n^ → H* is a mapping from the input space *R^n^* to a Hilbert space *H.* Red dots have labels *y_i_* = +1 while blue dots have labels *y_i_* = *-*1 in Figure 3(a) . And red dots have labels *y_i_* = 1, blue dots have labels *y_i_* = 2, while yellow dots have labels *y_i_* = 3 in Figure 3(b) and Figure 3(c) .

Now we give the detailed representation for our SVMHL model. The schematic plot of SVMHL is shown in Figure 3(c) . To illustrate our idea more clearly, we also plot the schemes for standard two class SVM and multi-class SVM in Figure 3(a) and Figure 3(b) side by side.

Given samples {*x_i_*, *y_i_*} for *i* = 1, ⋯ , *l*, where *x_i_* is a vector in the input space *R^n^* and *y_i_* denotes the corresponding class category taking a value in the output space {1, *⋯* , *q*}, where *q* is the total number of categories. In addition, the functional hierarchical tree, representing the relationships among these *q* categories, is known as the prior knowledge. The number of leaf nodes in the tree is *q.* A tree with *q* = 3 is shown in Figure 3(c) .

Suppose that, there are a total of *s* nodes in the hierarchical functional tree (In Figure 3(c), *s* = 5). To incorporate the inter-class relationships, we generate the SVM-based discriminant function to contain the contributions from all nodes along the paths from a root to a specific leaf node by introducing the class attribute vectors [5].

We introduce the attribute vector Λ(*y*) to characterize output label *y* by

Λ(*y*) = (*λ*_1_(*y*),*⋯* ,*λ_s_*(*y*))^T^, (1)

Where(2)

where *path*(*y*) is the category tags for the path from the root to the leaf node *y* in the functional hierarchical tree. For example, *path*(2) = {2, 5} in Figure 3(c) . Then the discriminant function becomes

*f*(*x*) = arg max*_y_*_∈{l_*_⋯_*_,_*_q_*_}_(w*·*Φ(*x*,*y*)), (3)

where  and(4)

where *ϕ*:*R^n^→H* is a mapping from the input space *R^n^* to a Hilbert space *H.*

A toy example is shown in Figure 3(c) to illustrate the discriminant function (3) generating procedure. The primal problem can be formulated as follows by incorporating the discriminant function (3):(5)

s*.*t*.* (w*·δ*Φ*_i_*(*y*)) ≽ 1*-ξ_i_*, *i* = 1,*···* ,*l*,*y* ≠ *y_i_*, (6)

*ξi* ≽ *0 i* = 1,⋯ ,*l*, (7)

where *δ*Φ*_i_*(*y*) = (w *·* Φ(*x_i_*,*y_i_*)) *-* (w *·* Φ(*x_i_*,*y*)).

The Lagrange dual optimization formulation of problem (5) ~ (7) becomes:(8)

s.t. *α_iy_* ≽ 0, *i* = 1, *⋯*,*l*, *y* ≠ *y_i_*, (9)(10)

The number of the variables equals *l ·* ( *q -* 1), the problem (8)~(10) will be quite large when *l* and *q* are large. For example, the number of leaf class labels is sixty-five and the number of proteins is five thousand and seven in the enzyme benchmark dataset. That is, *q* = 65 and *l* = 5, 007 in this dataset. The number of variables of problem (5) ~ (7) becomes 320, 448. Therefore, the computational challenge for this large scale optimization problem should be solved before applying it into the real-world problems.

From (5) ~ (7), we can find that, if a label  can make the following equations established(11)

that is,(12)

the inequality (6) will become . Then the primal problem (5) ~ (7) can be reformulated as follows:(13)(14)

*ξi* ≽ 0, *i* = 1,*⋯* ,*l*, (15)

where . So the Lagrange dual problem can be reformulated as follows:(16)

s.t. *α_i_* ≽ 0, *i* = 1,⋯ ,*l*, (17)

*α_i_* ≼ *C*, *i* = 1,⋯,*l.* (18)

The number of the variables for the problem (16) ~ (18) decreases to *l*. Note that, this problem is reduced to the standard SVM model by replacing the kernel function with . How to choose a suitable  for each input *x_i_*? The following Theorem provides some information for this task.

**Theorem 1.***Let k and l be the arbitrary two class labels in the leaf nodes and x be a test data point. α_iy_*, *i* = 1, *⋯* , *l* , *y ≠ yi are the optimal solutions of the problem* (8)*~*(10)*. Then for every pair of label tags k and l*, *we have the following equation*,(19)

Where(20)

**Proof:** w can be obtained by computing the derivations of lagrange function with respect to w, that is(21)

By using the mathematical expressions of *ϕ*(*x_i_*,*y_i_*), *ϕ*(*x_i_*,*y*) and |(w *·* Φ(*x*,*k*)) *-* (w *·* Φ(*x*,*l*))|^2^, we have(22)

By applying the Cauchy inequality the following inequality holds(23)(24)

Where(25)

Then the theory can be proved.

From the Theorem 1, the following Theorem can be deduced directly:

**Theorem 2.**The y* is the solution of the following problem if y* and y belong to the same superfamily.

y* = arg min*_y_*,_≠_*_y_*_,_*_y′_*,_∈{l,⋯__,_*_q_*_}_*Sup*(|(*w* • Φ(*x*, *y*)) *-* (w • Φ(*x*, *y′*))|)*.* (26)

where Sup is the least upper bound.

From the Theorem 2,  can be set to be one of the class labels belonging to the same superfamily of *y_i_.* The choice for  is introduced to put more emphasis on the class labels with same father to the  rather than the single class labels. Because the brother class labels are more easily to be confused by the predictive model. In addition, we let  be the class label belonging to the same super-family of *y_i_* with the largest proteins simultaneously to deal with the imbalance problem caused by the fact that the proteins in various enzyme function groups are constantly changing. This is because the class-boundary will be biased towards the large class heavily. For the same reason, if *y_i_* is a single class, and there is no class label as its brother, the corresponding  is set to be the class label with the largest scale.

### Implementing predictive model and evaluation criteria

By replacing the kernel function with , the freely available software for SVM implementation, LIBSVM (v.2.88) [26], can be used to solve our structure-based SVM model. The RBF kernel function is used here, and the penalty parameter *C* and the RBF kernel parameter *γ* are optimized by grid search approach with 3-fold cross-validation.

To evaluate the performance of our methods, we run the 10 fold cross-validation procedure. Besides for the accuracy, the Matthew’s correlation coefficient (MCC) [27] is used to further evaluate the performance of our method, which allows us to overcome the shortcoming of accuracy on imbalanced data [28].

## Authors contributions

YCW designed the predictive methods and the experiments, prepared the experiments and wrote the paper. YW analyzed the results and revised the paper. ZXY participated in developing the methods and revised the article. NYD proposed the idea for SVMHL.

## Competing interests

The authors declare that they have no competing interests.
